# Influence of Genetic Variance on Biomarker Levels After Occupational Exposure to 1,6-Hexamethylene Diisocyanate Monomer and 1,6-Hexamethylene Diisocyanate Isocyanurate

**DOI:** 10.3389/fgene.2020.00836

**Published:** 2020-08-19

**Authors:** Laura W. Taylor, John E. French, Zachary G. Robbins, Jayne C. Boyer, Leena A. Nylander-French

**Affiliations:** ^1^Department of Environmental Sciences and Engineering, Gillings School of Global Public Health, The University of North Carolina at Chapel Hill, Chapel Hill, NC, United States; ^2^Nutrition Research Institute, The University of North Carolina at Chapel Hill, Chapel Hill, NC, United States

**Keywords:** exposure assessment, biomarkers, biomonitoring, isocyanates, gene–environment interactions, genome-wide association study

## Abstract

We evaluated the impact of genetic variance on biomarker levels in a population of workers in the automotive repair and refinishing industry who were exposed to respiratory sensitizers 1,6-hexamethylene diisocyanate (HDI) monomer and one of its trimers, HDI isocyanurate. The exposures and respective urine and plasma biomarkers 1,6-diaminohexane (HDA) and trisaminohexyl isocyanurate (TAHI) were measured in 33 workers; and genome-wide microarrays (Affymetrix 6.0) were used to genotype the workers’ single-nucleotide polymorphisms (SNPs). Linear mixed model analyses have indicated that interindividual variations in both inhalation and skin exposures influenced these biomarker levels. Using exposure values as covariates and a false discovery rate < 0.10 to assess statistical significance, we observed that seven SNPs were associated with HDA in plasma, five were associated with HDA in urine, none reached significance for TAHI in plasma, and eight were associated with TAHI levels in urine. The different genotypes for the 20 significant SNPs accounted for 4- to 16-fold changes observed in biomarker levels. Associated gene functions include transcription regulation, calcium ion transport, vascular morphogenesis, and transforming growth factor beta signaling pathway, which may impact toxicokinetics indirectly by altering inflammation levels. Additionally, in an expanded analysis using a minor allele cutoff of 0.05 instead of 0.10, there were biomarker-associated SNPs within three genes that have been associated with isocyanate-induced asthma: *ALK*, *DOCK2*, and *LHPP*. We demonstrate that genetic variance impacts the biomarker levels in workers exposed to HDI monomer and HDI isocyanurate and that genetics can be used to refine exposure predictions in small cohorts when quantitative personal exposure and biomarker measurements are included in the models.

## Introduction

Isocyanates are one of the leading causes of occupational asthma around the world (Malo and Chan-Yeung, 2009; Dao and Bernstein, 2018). A wide range of isocyanate-induced asthma prevalence has been reported in various industries, with some estimates as low as < 1% and others as high as > 30% (Lockey et al., 2015). The three main isocyanates produced are toluene diisocyanate (TDI), methylene diphenyl diisocyanate (MDI), and 1,6-hexamethylene diisocyanate (HDI) (Randall and Lee, 2002). Of those, HDI is primarily used in automotive industries. The American Conference of Governmental Industrial Hygienists’ threshold limit value–time weighted average (TLV-TWA) for HDI monomer is 34 mg/m^3^ (ACGIH, 2010), and it has been estimated that it only takes ∼6.5 min of spray painting for workers to achieve a dose equivalent to the TLV-TWA (Thomasen and Nylander-French, 2012). No TLV has been set for HDI isocyanurate exposure in the workplace because less research has been conducted on the oligomers of HDI even though the oligomers are measured at greater concentrations than HDI monomer in the personal breathing zone and/or on the skin of spray painters in the automotive refinishing industry (Woskie et al., 2004; Pronk et al., 2006a,b; Bello et al., 2008; Fent et al., 2009a,b; Reeb-Whitaker et al., 2012).

Because only a subset of workers develops occupational asthma from exposure to isocyanates, genetic variability is thought to impact workers’ susceptibility to developing isocyanate-induced asthma. Multiple studies have reported genetic markers associated with risk of developing isocyanate-induced occupational asthma. Among these studies, susceptibility and/or protective genetic markers in the human leukocyte antigen (*HLA*), glutathione *S*-transferase (*GST*), alpha catenin (*CTNNA*), cadherin (*CDH*), *N*-acetyltransferase (*NAT*), and Krüppel C2H2-type zinc-finger (*ZBTB*) gene families have been reported in two or more studies (Mapp et al., 2000; Piirilä et al., 2001; Wikman et al., 2002; Choi et al., 2009; Kim et al., 2009; Yucesoy et al., 2012, 2014, 2015a; Bernstein et al., 2013, 2018). These genes have functions including antigen presentation, xenobiotic metabolism, ell–cell adhesion, and inflammatory response (Mapp et al., 2000; Piirilä et al., 2001; Wikman et al., 2002; Choi et al., 2009; Kim et al., 2009; Yucesoy et al., 2012, 2014; Bernstein et al., 2013).

Little is known about the pathways involved in the absorption, distribution, metabolism, and elimination (ADME) of isocyanates before asthma pathology develops. Research on protein adducts and asthma risk indicates that GST, NAT, and/or CYP450 gene products may be involved in the biotransformation and metabolism of isocyanates (Flack et al., 2010a), but this still needs to be researched further. The impact of genetics on isocyanate metabolite levels after occupational exposure has been investigated in a few studies. Broberg et al. (2010) found that a polymorphism in a metabolism gene *GSTP1* Ile105 Val was correlated with differences in TDI biomarker levels in plasma and urine. More recently, it was observed that two SNPs in *P2RX7* modified the relationship between TDI urine biomarker levels and plasma levels of lysophosphatidic acid, which may affect the inflammatory response after isocyanate exposure (Broström et al., 2018).

To help bridge the knowledge gap about what causes the observed differences in workers’ biomarker levels after HDI monomer and HDI isocyanurate exposures, we examined the influence of interindividual genetic differences on biomarker levels after occupational exposure for both HDI monomer and HDI isocyanurate. Increased knowledge about the impact of interindividual differences in genetics on isocyanate biomarker levels could help to refine exposure models to these chemicals and improve understanding of the toxicokinetics of isocyanate exposures and isocyanate-associated adverse health outcomes.

## Materials and Methods

### Study Participants

The characteristics of the full study population have been previously described (Fent et al., 2009a,b; Gaines et al., 2010a; Flack et al., 2010b). In summary, 33 automotive spray painters from the Greater Triangle area in North Carolina and Puget Sound area in Washington provided complete HDI monomer and isocyanurate exposure and genetic data and were included in this genome-wide association study (GWAS). The 33 participants were all male with an average age of 35 ± 9 years (range: 21–59 years), were predominantly of Caucasian descent (25 of the 33) with an average of 13 ± 10 years (range: 0.5–35 years) in spray painting. The study was approved by The University of North Carolina at Chapel Hill Office of Human Research Ethics Institutional Review Board (Study #12-1195) and by the Washington State Department of Social and Health Services Institutional Review Board (Study #A-013106).

### Assessment of Exposure and Biomarker Levels

Sampling and quantification of inhalation and skin exposures to HDI monomer and its oligomers including HDI isocyanurate as well as biomarkers of exposure have been published previously (Fent et al., 2009a,b; Flack et al., 2010b, 2011; Gaines et al., 2010a; Robbins et al., 2018). Briefly, exposure and biomarker levels were measured for consenting spray painters who were visited during one workday, with up to three visits during the span of a year to increase statistical power, spaced at least 3 weeks apart. By measuring urine and blood biomarkers of isocyanate exposure using the repeated sampling protocol, we can obtain more robust data to elucidate the effect of intraindividual and interindividual variability for correlation with personal and external factors that may modify the internal dose received by the individual (Rappaport et al., 1995; Lyles et al., 1997; Serdar et al., 2006). The interval of 3 weeks was chosen to reduce the potential for biomarker measurements being confounded by exposure received during the previous sampling visit considering that (1) the half-life of albumin is 3 weeks (Peters, 1970) and (2) tape-stripping depletes the stratum corneum and may affect barrier function, therefore, we allowed sufficient time for skin rejuvenation (Grove and Kligman, 1983; Takahashi et al., 1987).

HDI monomer and HDI isocyanurate inhalation and skin exposures were quantified during each paint task using personal breathing zone and skin tape-strip sampling (Fent et al., 2009a,b). Filter cassettes were used to capture air samples from the workers’ breathing zone; and HDI monomer and HDI isocyanurate levels were analyzed using high-performance liquid chromatography–mass spectrometry (HPLC-MS) (Fent et al., 2009a). Time-weighted average personal breathing-zone concentration (TWA; μg/m^3^) was adjusted with the Occupational Safety and Health Administration (OSHA) assigned protection factor (APF) based on respirator type (Occupational Safety and Health Administration, 2009) and then multiplied by the time spent painting (min) and the average male breathing rate (0.0232 m^3^/min) to estimate the daily inhalation exposure (μg) (Adams, 1993). Skin exposure to HDI monomer and to HDI isocyanurate was evaluated by using HPLC-MS on tape-strip samples as described previously (Fent et al., 2009b). Briefly, tape-strips were collected on the dorsal side of each hand and on the dorsal and volar side of each lower arm. Neck and wrists were also sampled when workers were not wearing hood respirators and when coveralls/gloves were not covering the wrists, respectively. Skin exposure was estimated as a mass of exposure (μg) by calculating the sum of HDI monomer or HDI isocyanurate in the tape strips collected from the sampled body parts.

The biomarkers used were the hydrolyzed products of HDI monomer and HDI isocyanurate, 1,6-diaminohexane (HDA), and trisaminohexyl isocyanurate (TAHI), respectively. For assessment of biomarker levels in urine, every urine void was collected during the full workday for each visit, and isocyanate levels were quantified for each urine sample using gas chromatography–mass spectrometry (GC-MS) for HDA (Gaines et al., 2010a) and using nanoflow ultra-performance liquid chromatography coupled with nano-electrospray ionization tandem mass spectrometry (nano-UPLC-ESI-MS/MS) for TAHI (Robbins et al., 2018). Each urine sample’s biomarker level was adjusted with its creatinine level (μg/g creatinine) (Gaines et al., 2010b). For plasma biomarker assessment, one blood sample was drawn near the end of each visit and was evaluated using GC-MS for HDA (Flack et al., 2010b) and nano-UPLC-ESI-MS/MS for TAHI (Robbins, 2019). Plasma level (μg) was calculated by multiplying the plasma biomarker concentration (μg/L) by the worker’s plasma volume (L), which was estimated using the individual’s body weight with a male-specific factor of 0.04 L/kg (American Association of Blood Banks, 1996).

For statistical analyses in SAS, all exposure and biomarker levels were natural log-transformed to improve normality and stabilize the variance. Prior to natural log-transformation, exposure and biomarker levels below the method detection limit (MDL) or limit of detection (LOD) were imputed to non-zero values using the equation (MDL/$\sqrt{2}$)/100 or (LOD/$\sqrt{2}$)/100 to help satisfy normality assumption for statistical analyses. The geometric mean of daily inhalation and skin exposures was calculated across all of the visits for each spray painter for analysis in PLINK v2.0, an open source genome association analysis toolset (Chang et al., 2015), because PLINK is unable to accommodate multiple measurements of a single variable for each subject. For urine biomarker levels, the geometric mean of the average creatinine-adjusted concentration of all voids during each visit was calculated for every worker. For plasma biomarker levels, the geometric mean was calculated across all visits for every worker.

### Genotyping and Quality Control Procedures

Whole blood samples were collected in tripotassium ethylenediaminetetraacetic acid (K_3_EDTA) anticoagulant tubes for genotyping. The peripheral blood mononuclear cells (PBMCs) were separated from whole blood using Ficoll^TM^ separation and then QIAAmp Blood mini kits (Qiagen, Germantown, MD, United States) were used to isolate DNA from the PBMCs. The DNA was quantified using a NanoDrop spectrophotometer (Thermo Fisher Scientific, Waltham, MA, United States), and diluted to 50 ng/μl using 10 mM of Tris, pH 7.4. For 10 of the worker’s samples, Qiagen REPLI-g genomic amplification kit was used. DNA was digested using restriction enzymes, purified, ligated to Affymetrix single-nucleotide polymorphism (SNP) 6.0 Core Reagent Kit adaptors, and amplified with generic primers for the adaptor sequence using polymerase chain reaction (PCR). Afterward, the DNA fragments were hybridized to the Affymetrix Genome-wide Human SNP Array 6.0 (Affymetrix, Santa Clara, CA, United States) featuring more than 906,600 SNPs.

Genotyping was carried out for the 33 workers in this study plus an additional 23 workers with similar worker characteristics from an intervention study that was performed by the same researchers (Fletcher, 2015; Kim, 2015). The additional 23 workers were an average of 40 ± 10 years old (range: 24–64 years old); 22 were Caucasian and had been painting for 18 ± 9 years (range: 3–35 years). Genotyping data (*n* = 56) was cleaned using PLINK v2.0. For quality control (QC), only individuals with < 10% missing SNP data were included. Only autosome markers that did not fail the Hardy–Weinberg departure with *p*< 0.001, were successfully genotyped at a rate > 90%, and had a minor allele frequency (MAF) of *q* > 0.10 were included in the final data set. SNPs from the X chromosome were not included in the cleaned data because of QC issues with those markers. A total of 533,673 SNPs passed QC. Note that the two study populations with exposure to the same compounds in the same occupational settings were combined only for genotyping QC cleaning to increase the threshold to make an accurate genotype call.

### Genome-Wide Association Study Using Multiple Linear Regression

Genotyping data for the workers with full genotyping and exposure data (*n* = 33) were analyzed using PLINK v2.0. Genetic marker associations with HDA and TAHI biomarker levels were investigated using multiple linear regression analysis. The biomarker levels were inputted as the phenotype, and the measured inhalation and skin exposure levels were inputted as covariates. Multidimensional scaling (MDS) methods were used to determine that neither smoking nor ethnicity was a contributing covariate to the reported results. Elimination of smoking and ethnicity as covariates was determined by calculating identity by state (IBS) distances and MDS clustering with 10 dimensions in PLINK and viewing the plots using PROC SGPLOT in Statistical Analysis System (SAS) 9.4 (SAS Institute, 2017). Genetic markers were considered to be statistically significant at a false discovery rate (FDR) < 0.10. This was tighter than Efron’s recommended minimum cutoff of 0.20 (Efron, 2007), in which FDR is a statistical test that aims to reduce the type I error rate (false positives) when making multiple comparisons (Benjamini and Hochberg, 1995) and can be used for GWAS (Bush and Moore, 2012).

All statistically significant SNPs detected by PLINK using summary exposure and biomarker values were confirmed in SAS using all of the measurements of exposure and biomarker levels during every visit. R version 3.4.4 (RStudio Team, 2015) was used to generate Manhattan plots (manhattan command in the qqman package), with default suggestive and genome-wide significance lines set at *p* = 1 × 10^–5^ and *p* = 5 × 10^–8^, respectively (Turner, 2018). We also added a third line at *p* = 1 × 10^–6^, where FDR∼0.10 for our study. LocusZoom was then used to examine regions with significant SNPs in greater detail (Pruim et al., 2010). R statistical ggplot2 package was used to generate box and whisker plots of biomarker variance by SNP allele for the significant markers using the qplot command (Wickham, 2016). An additive model of polygenic inheritance was used to evaluate the fold-differences in average biomarker levels among workers with different genotypes for each SNP, and then the average fold-difference and 95% confidence interval between genotypes were calculated across all 20 SNPs. SAS version 9.4 was used to calculate *R*^2^ (PROC GLM command) for each significant SNP’s alleles and the unadjusted isocyanate biomarker levels with which the SNP was associated. The average *R*^2^ and 95% confidence interval were then averaged for all 20 significant SNPs.

### Linear Mixed-Effects Exposure Model

Linear mixed-effects models with compound symmetry were used to assess the levels of contribution of inhalation exposure, skin exposure, and the SNP with statistically the strongest association with HDA or TAHI biomarker levels (PROC MIXED command in SAS). A *p*-value cutoff of < 0.10 was used to determine statistical significance. The model included natural log-transformed data (from every visit for each worker) in order to satisfy normality assumption for statistical analyses. Association between the most significant SNP and the biomarker levels was determined by fitting the models for HDA in plasma, HDA in urine, TAHI in plasma, and TAHI in urine for the *i*^*th*^ worker at the *v*^*th*^ visit. *H_iv_* represents the HDI biomarker levels (either HDA or TAHI) in plasma or urine for the *i*^*th*^ individual worker at their *v*^*th*^ visit, *R*_*ijr*_ represents the *r*^*th*^ isocyanate inhalation exposure level for the *i*^*th*^ worker at the *v*^*th*^ visit, *S*_*ijs*_ represents the *s*^*th*^ isocyanate skin exposure level for the *i*^*th*^ worker on their *v*^*th*^ visit, *G*_*ig*_ represents the *g*^*th*^ genetic marker for the *i*^*th*^ worker, α*_*i*_* represents the random effects associated with the *i*^*th*^ individual, *ε_*i*__*v*_* represents the random errors associated with the *v*^*th*^ visit for the *i*^*th*^ individual, β_0_ is the slope, and β*_*r*_*, β*_*s*_*, and β*_*g*_* represent the corresponding regression coefficients. The model was:

$$\ln\left(H_{i\,v}\right)=\beta_{0}+\beta_{r}\,\ln\left(R_{i\,v\,r}\right)+\beta_{s}\,\ln\left(S_{i\,v\,s}\right)+\beta_{g}\,G_{i\,g}+\alpha_{i}+\varepsilon_{i\,v}$$

### Bioinformatics Analysis

The proprietary Affymetrix nomenclature for SNP alleles were converted to reference SNP cluster identification (rsID) numbers using the January 2017 annotation file from Affymetrix for the Genome Wide Human SNP Array 6.0 (available under support files for catalog item 901153 on www.thermofisher.com). SNP positions were lifted from GRCh37/hg19 to the GRCh38/hg38 genome assembly. The statistically significant SNPs were searched in the SNP database (dbSNP) from the National Center for Biotechnology Information (NCBI) database using their rsIDs to identify proximal coding genes and regulatory elements. In order to identify and explore potential network interactions on the basis of the significant SNPs, this set of genes was then input into two bioinformatics resources: the Database for Annotation, Visualization and Integrated Discovery (DAVID) v6.8, and GeneMANIA. DAVID uses a single-linkage method to agglomerate gene and protein identifiers from many public bioinformatics resources to display information including gene ontology terms, molecular functions, functional annotation clustering, and gene similarity (Sherman et al., 2007). GeneMANIA uses a label propagation algorithm to predict direct and indirect network interactions based on validated unbiased protein–protein and protein–DNA interactions (Warde-Farley et al., 2010; Zuberi et al., 2013). For this study, GeneMANIA was used to search for co-expression, co-localization, genetic interactions, pathways, physical interactions, predicted networks, and shared protein domains using the molecular function setting for the gene ontology weighting. Literature searches were also conducted for each gene and regulatory element separately in the NCBI databases dbSNP and PubMed to learn more about their respective functions.

### Candidate-Gene Analysis

Candidate genes identified from a curated scientific literature search were used to evaluate whether the identified biomarker-associated SNPs were within genes that have been associated with isocyanate-induced asthma in the published literature. The list of biomarker-associated SNPs was expanded for this analysis, using a SNP MAF cutoff of > 0.05 instead of a MAF > 0.10 in order to decrease the risk of false negatives. A total of 63 candidate genes were evaluated. Of those, 55 were directly from previously published papers on isocyanate-asthma: *ACMSD*, *AHNAK*, *ALK*, *ASTN2*, *ATF3*, *C11orf74*, *CDH17*, *CRTAC1*, *CTNNA1*, *CTNNA3*, *DOCK2*, *EPHX1*, *FAM71A*, *GADL1*, *GSTM1*, *GSTP1*, *HERC2*, *HLA-A*, *HLA-B*, *HLA-C*, *HLA-DOA*, *HLA-DPB1*, *HLA-DQA1*, *HLA-DQA2*, *HLA-DQB1*, *HLA-DRB1*, *HLA-E*, *IBTK*, *IL4RA*, *KCNIP4*, *LHPP*, *MBL2*, *NAT1*, *NAT2*, *NPAS3*, *ODZ3*, *PCNX*, *PCTK2*, *PDGFD*, *PFKFB3*, *PITPNC1*, *PRKCA*, *PTGS1*, *PTGS2*, *SAMD12*, *SLC24A2*, *SLC6A12*, *SOD2*, *TACR1*, *TGF*β*1*, *TNF*α, *TRPM8*, *TUSC3*, *UGT2B4*, and *ZBTB16* (Bernstein, 2011; Yucesoy et al., 2012, 2014, 2015a,b, 2016; Hur and Park, 2015; Bernstein et al., 2018). An additional eight candidate genes were added to the list after lifting positions for asthma-associated SNPs to the newest genome build, GRCh38/hg38. The eight additional genes were *FBXL7*, *GSTM3*, *GSTM5*, *PACERR*, *PCNX1*, *PDZRN4*, *TENM3*, and *TMEM91*. Linkage disequilibrium (LD) between our biomarker-associated SNPs in the candidate genes and the reported asthma-associated SNPs in the candidate genes was evaluated using a tool from the National Institutes of Health (NIH)^1^.

## Results

### Genome-Wide Association Study

**FIGURE 1:**
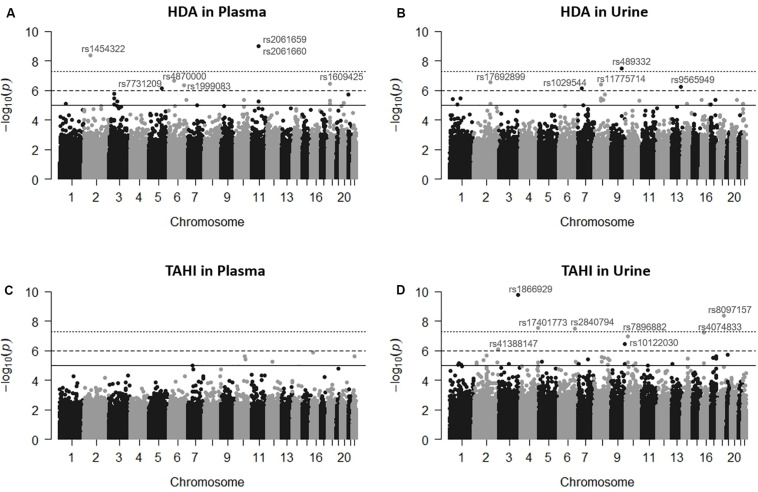
Manhattan plots showing the single-nucleotide polymorphisms (SNPs) significantly associated with 1,6-diaminohexane (HDA) in plasma **(A)** and urine **(B)** and trisaminohexyl isocyanurate (TAHI) biomarker levels in plasma **(C)** and urine **(D)** when inhalation and skin exposures to 1,6-hexamethylene diisocyanate (HDI) monomer and HDI isocyanurate were used as covariates, respectively. Urine biomarker levels were adjusted with creatinine level. The solid line is the suggestive significance default line at -log_10_(*p* = 1 × 10^–5^), the dashed line is equivalent to false discovery rate (FDR) = 0.10 at −log_10_(*p* = 1 × 10^–6^), and the top dotted line is the genome-wide significance default line at −log_10_(*p* = 5 × 10^–8^).

Manhattan plots revealed that the variation in HDI monomer and HDI isocyanurate biomarker levels (i.e., HDA and TAHI, respectively) was explained in part by the interindividual genetic marker variation (Figure 1). GWAS analysis in PLINK, using exposure to HDI monomer or HDI isocyanurate as covariates and FDR < 0.10 for significance, revealed that seven SNPs were associated with HDA levels in plasma, five SNPs were associated with HDA levels in urine, and eight SNPs were associated with TAHI levels in urine (Table 1). No SNP associations reached significance for TAHI levels in plasma (Supplementary Table S1). In general, inspection of the LocusZoom plots showed that sentinel SNPs in LD with the 20 significant SNPs had higher statistical significance than nearby SNPs that were not in LD (Supplementary Figures S1–S3).

**TABLE 1:** Genes proximal to the 20 single-nucleotide polymorphisms (SNPs) [minor allele frequency (MAF) > 0.10] significantly associated with differences in isocyanate plasma and urine biomarker levels when adjusted for inhalation and skin exposure covariates.

**SNP**	**Chr**	**Position**	**MAF**	***p*-value**	**FDR**	**Proximal genes (SNP location)**
**(A) SNPs associated with HDA in plasma**						
rs2061660	11	23385393	0.17	1.08E-09	0.000289	*LOC100131557* and *WIZP1* (intergenic)
rs2061659	11	23385468	0.17	1.08E-09	0.000289	*LOC100131557* and *WIZP1* (intergenic)
rs1454322	2	157659909	0.04	3.63E-09	0.000646	*ACVR1C*, *ACVR1*, and *LOC105373713* (intergenic)
rs4870000	6	151215650	0.14	2.21E-07	0.0295	*LOC102723831* (intronic)
rs1609425	18	36765721	0.09	3.66E-07	0.0391	*FHOD3* (intronic)
rs1999083	6	71740877	0.15	4.69E-07	0.0417	*RNU4-66P*, *KRT19P1*, and *RIMS1* (intergenic)
rs7731209	5	37816743	0.15	7.44E-07	0.0567	*GDNF* (intronic)
**(B) SNPs associated with HDA in urine**						
rs489332	9	75413430	0.18	3.07E-08	0.0164	*LOC107987080* and *LOC105376091* (intergenic)
rs17692899	2	29062388	0.07	2.74E-07	0.0696	*PCARE* (intronic)
rs11775714	8	16050222	0.05	3.91E-07	0.0696	*RPL32P19* and *MSR1* (intergenic)
rs9565949	13	84630746	0.15	5.70E-07	0.076	*LINC00333* and *LOC105370289* (intergenic)
rs1029544	7	14084440	0.23	7.36E-07	0.0785	*ETV1* and *DGKB* (intergenic)
**(C) SNPs associated with TAHI in urine**						
rs1866929	3	73865896	0.13	1.64E-10	8.73 × 10^–5^	*LINC02005* (intronic), *PDZRN3* and *CNTN3* (intergenic)
rs8097157	18	6133403	0.2	3.91E-09	0.00104	*L3MBTL4* (intronic)
rs17401773	4	94947147	0.07	2.67E-08	0.0044	*BMPR1B* (intronic)
rs2840794	6	72749667	0.2	3.29E-08	0.0044	*KCNQ5* (intronic)
rs4074833	16	51641880	0.38	6.01E-08	0.00642	*LOC102723323*, *LOC105371257*, and *SALL1* (intergenic)
rs7896882	10	119450311	0.22	1.09E-07	0.00968	*GRK5* (intronic)
rs10122030	9	93591220	0.18	3.38E-07	0.0257	*PHF2* (intronic)
rs41388147	2	81967239	0.13	8.42E-07	0.0562	*RN7SL201P* (intronic)

**SNPs with an FDR < 0.10 were considered statistically significant. Chr, chromosome; HDA, 1,6-diaminohexane; TAHI, trisaminohexyl isocyanurate.**

**FIGURE 2:**
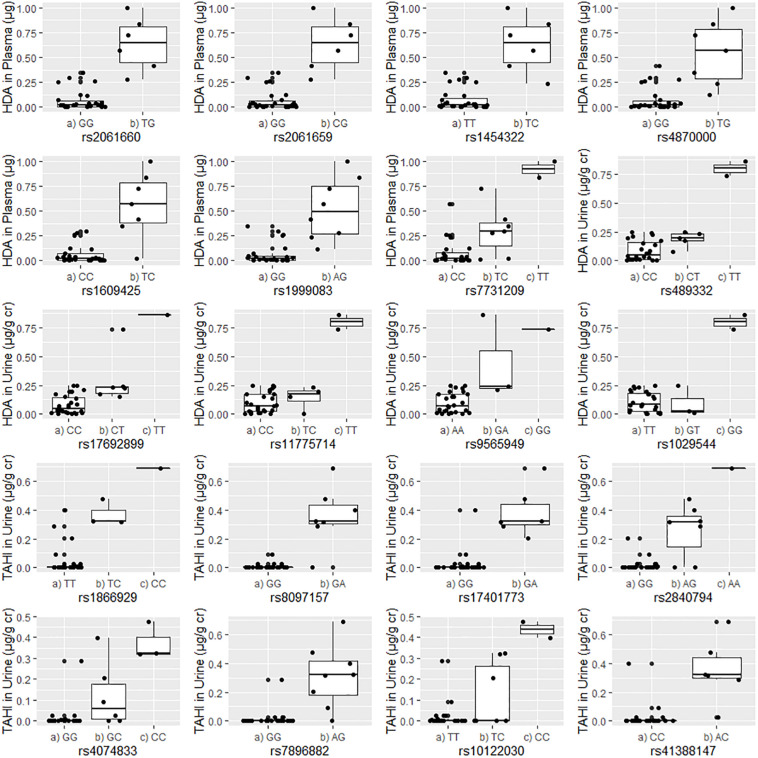
Correlations between the unadjusted biomarker levels in urine or plasma and the genotypes for each of the 20 significant single-nucleotide polymorphisms (SNPs). Urine biomarker levels were adjusted with creatinine level (μg biomarker/g creatinine; μg/g Cr). The homozygous major allele is preceded by “(a),” the heterozygous genotype is preceded by “(b),” and the homozygous minor allele is preceded by “(c).”

Boxplots of unadjusted biomarker concentrations versus alleles for the 20 significant SNPs are shown in Figure 2. The rare homozygous genotype was present for 10 of the significant SNPs. *R*^2^-values for the boxplots showed that the SNP alleles were correlated well with biomarker levels, with *R*^2^ ranging from 0.60 to 0.85 with an average of 0.71 ± 0.07 (data not shown). For the 20 significant SNPs, workers who were homozygous for the minor allele had an average of four times higher biomarker concentrations than workers with the heterozygous genotype (95% confidence interval: 2- to 5-fold difference), and an average of 16 times higher biomarker concentrations in plasma and urine than workers who were homozygous for the major allele (95% confidence interval: 8- to 23-fold difference). Workers who had a heterozygous genotype had an average of 10 times higher biomarker concentration than workers who were homozygous for the major allele (95% confidence interval: 6- to 14-fold difference).

### Linear Mixed-Effects Models

With the use of a *p*-value of 0.10 for significance, mixed models that were run with and without the most significant SNP for each biomarker and media (i.e., plasma and urine) indicated that both isocyanate inhalation exposure and skin exposure were correlated with biomarker levels. For HDA biomarker levels (Supplementary Table S2), skin exposure to HDI monomer was significantly correlated with HDA in plasma (*p* = 0.076) and in urine (*p* = 0.003), but inhalation exposure was not significant in either model. When the most significant SNP was added to the models (rs2061660 for HDA in plasma and rs489332 for HDA in urine), the pattern of HDI monomer skin exposure being significant while inhalation exposure being insignificant remained the same for HDA urine levels. However, that was not the case for HDA plasma levels: inhalation exposure to HDI monomer was significant (*p* = 0.086) whereas skin exposure lost significance. Neither inhalation nor skin exposure was influential for TAHI levels in plasma. However, skin and inhalation exposures to HDI isocyanurate were both significantly correlated with TAHI levels in urine (*p* = 0.078 and *p* = 0.047, respectively), which remained true when the most significant SNP for TAHI in urine, rs1866929, was added to the model (Supplementary Table S3).

### Bioinformatics

The functions and predicted interaction networks for the protein products of the genes that contain or are proximal to the most significant biomarker-associated SNPs were similar for HDA and TAHI. The predicted protein network functions were suggestive of the importance of the TGF-β pathway, gene expression modulation, cell migration, and calcium regulation for affecting HDA and TAHI biomarker levels.

**FIGURE 3:**
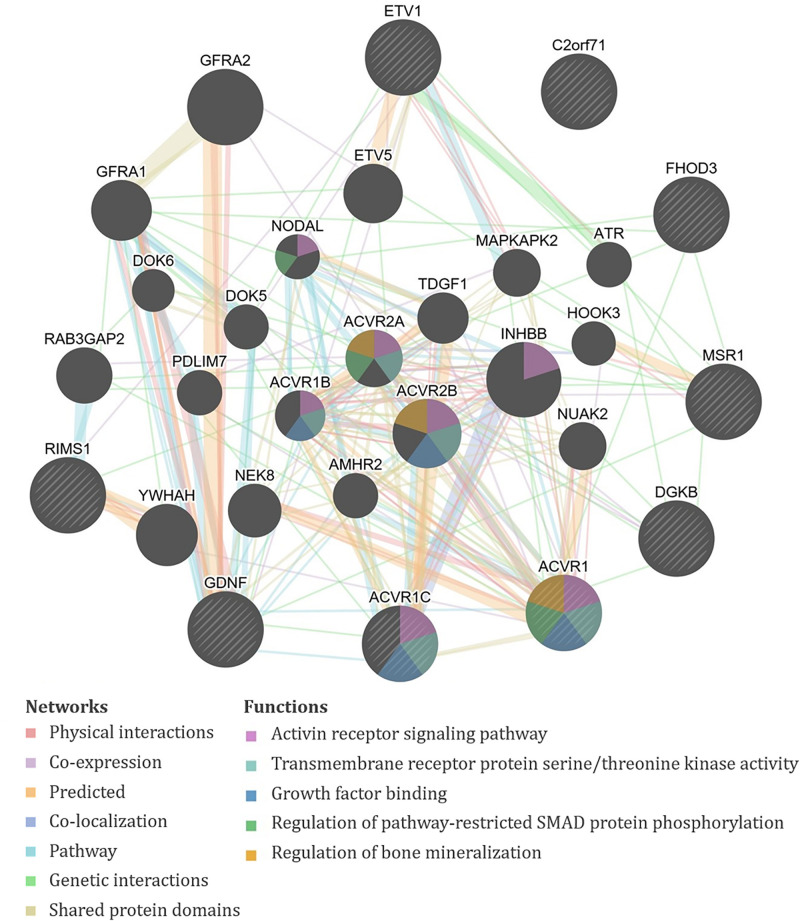
GeneMANIA output for the products of genes proximal to the significant single-nucleotide polymorphisms (SNPs) associated with exposure-adjusted 1,6-diaminohexane (HDA) levels in plasma and urine. Query genes have black circles with white-striped lines, the networks are shown with the colored lines between genes, and the shading on the circles shows the functions.

Specifically, the seven significant SNPs for HDA in plasma were within or proximal to the genes *ACVR1*, *ACVR1C*, *FHOD3*, *GDNF*, *KRT19P1*, *RIMS1*, *RNU4-66P*, and *WIZP1* (Table 1). There were non-coding RNAs (ncRNAs) and pseudogenes that may be impacted by the significant SNPs as well, including LOC100131557, which is a programmed cell death 2-like pseudogene. The genes proximal to the five significant SNPs for HDA in urine included *ETV1*, *DGKB*, *MSR1*, *PCARE* (formerly *C2orf71*), and *RPL32P19* (Table 1). GeneMANIA showed that there were overlapping networks for all HDA-biomarker related genes that it has in its database (*ACVR1*, *ACVR1C*, *ETV1*, *DGKB*, *FHOD3*, *GDNF*, *MSR1*, and *RIMS1*) except for *PCARE/C2orf71* (Figure 3). The main pathways predicted by GeneMANIA for HDA-biomarker related genes’ protein products included activin receptor signaling pathway, transmembrane receptor protein serine/threonine kinase activity, growth factor binding, regulation of bone mineralization, and regulation of pathway-restricted SMAD protein phosphorylation (Figure 3).

**FIGURE 4:**
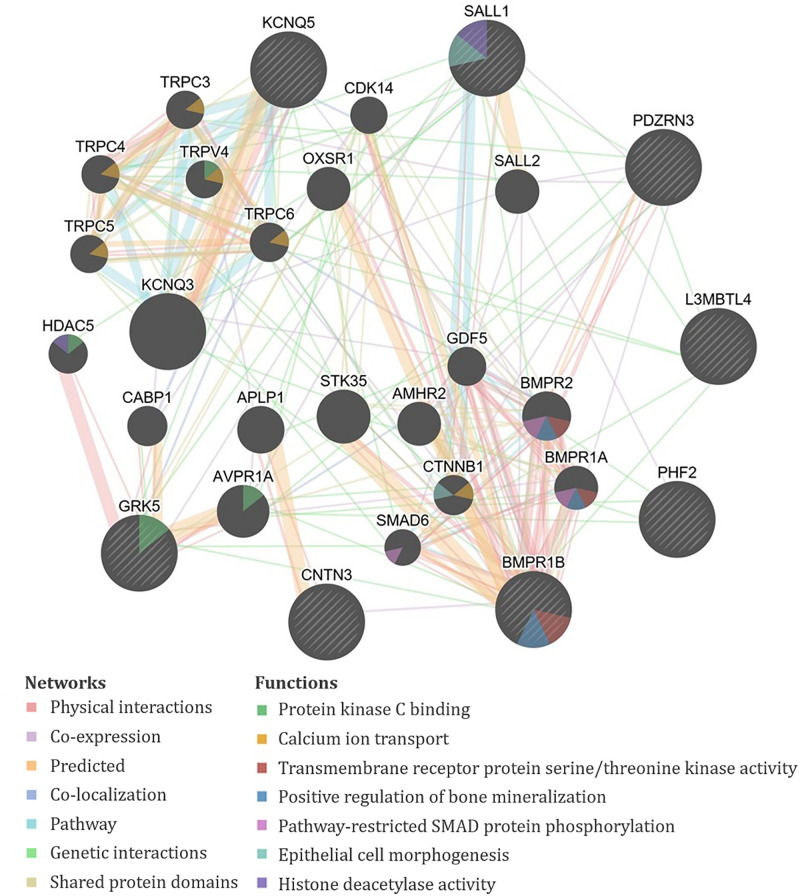
GeneMANIA output for the products of genes proximal to the significant single-nucleotide polymorphisms (SNPs) associated with exposure-adjusted trisaminohexyl isocyanurate (TAHI) levels in urine. Query genes have black circles with white-striped lines, the networks are shown with the colored lines between genes, and the shading on the circles shows the functions.

The genes containing or proximal to the eight significant SNPs for TAHI in urine included *BMPR1B*, *CNTN3*, *GRK5*, *KCNQ5*, *L3MBTL4*, *PDZRN3*, *PHF2*, *RN7SL201P*, and *SALL1* (Table 1). All of the TAHI-associated genes that were recognized by GeneMANIA (*BMPR1B*, *CNTN3*, *GRK5*, *KCNQ5*, *L3MBTL4*, *PDZRN3*, *PHF2*, and *SALL1*) had overlapping networks that included physical and genetic interactions, co-expression, and co-localization (see Figure 4). Some of the main pathways predicted by GeneMANIA for the gene’s protein products included protein kinase C binding, calcium ion transport, transmembrane receptor protein serine/threonine kinase activity, BMP signaling, regulation of bone mineralization, SMAD protein phosphorylation, epithelial cell morphogenesis, and histone deacetylase activity (Figure 4).

When comparing the structure of the genes in DAVID, two genes associated with HDA in plasma, *ACVR1* and *ACVR1C*, were highly related to one of the genes associated with TAHI in urine, *BMPR1B* (kappa = 0.51, high similarity). All three genes are included in the transforming growth factor-beta (TGF-β) superfamily of structurally related signaling proteins (Chida et al., 2012; Ehrlich et al., 2012; Schwartze et al., 2014; Kim et al., 2016).

### Candidate-Gene Analysis

None of the 20 biomarker-associated SNPs with the more stringent cutoff of MAF > 0.10 were within the candidate genes associated with isocyanate-asthma. When evaluating SNPs with a lower threshold of MAF > 0.05, instead of 20 significant SNPs, there were 643 SNPs that were associated with isocyanate biomarker levels (FDR < 0.10). When we evaluated whether those 643 SNPs were within any of the 55 genes reported in the literature as being associated with isocyanate-induced asthma, four SNPs were within three genes that were reported by Kim and colleagues (Table 2) in their GWAS study published in 2009 (Kim et al., 2009). The genes were *ALK*, *DOCK2*, and *LHPP* (Kim et al., 2009). The biomarker-associated SNPs within those genes from our study were not in LD with any of the asthma-associated SNPs that Kim and colleagues reported within those genes.

**TABLE 2:** Three candidate genes contained four single-nucleotide polymorphisms (SNPs) [minor allele frequency (MAF) > 0.05] that were associated with exposure-adjusted biomarker levels [false discovery rate (FDR) < 0.10].

**Gene**	**Chr**	**SNPs associated with isocyanate biomarker levels**						SNPs associated with isocyanate asthma*			
		**SNP**	**Position**	***p*-value**	**FDR**	**Biomarker**	**Media**	**SNP**	**Position**	***p*-value**	**OR (LCI**–**UCI)**
*ALK*	2	rs6731230	29263212	5.80E-07	0.00635	TAHI	Urine	rs7599783	29310916	3.97E-05	3.43 (1.84–6.40)
								rs7599686	29310870	4.16E-05	3.40 (1.82–6.34)
								rs4666199	29312331	6.44E-05	3.38 (1.81–6.30)
*DOCK2*	5	rs12522080	169922991	4.49E-06	0.06886	HDA	Plasma	rs10054135	169832121	6.49E-05	3.28 (1.79–5.99)
		rs12515095	169878149	5.34E-07	0.00838	HDA	Urine				
*LHPP*	10	rs11598732	124534942	1.24E-05	0.05001	TAHI	Urine	rs4962619	124486739	6.33E-05	3.67 (1.88–7.14)

Chr, chromosome; LCI, lower 95% confidence interval; OR, odds ratio (specifically, for developing isocyanate asthma compared with normal health controls); UCI, upper 95% confidence interval. * All of the intragenic SNPs that were associated with isocyanate asthma were reported by Kim et al. (2009). SNP positions were lifted to build GRCh38.

## Discussion

### Genome-Wide Association Study

There were 20 SNPs that were associated with HDA and TAHI biomarker levels following workers’ inhalation and skin exposures to paints containing HDI monomer and HDI isocyanurate. Workers who were homozygous for the minor allele for the 20 SNPs had an average of 4–16 times higher biomarker concentration in plasma and urine than workers who were heterozygous or homozygous for the major allele (*R*^2^ for boxplots were 0.71 ± 0.07). Exposure assessment relying only on monitoring of biomarker levels would result in an overestimation of workers’ external exposure (i.e., inhalation and skin exposures) by 4- to 16-fold in individuals who are heterozygous or homozygous for the minor allele for these SNPs, respectively. Furthermore, the significant SNPs discovered in this study are all relatively common in the population, with an intrapopulation MAF > 10% and an average global MAF of 15% occurrence in the 1000 Genomes phase 3 project genotype data^2^, suggesting that incorporating these SNPs into exposure modeling would help reduce error in exposure assessment.

The potential significance of workers who were homozygous for the minor allele having higher biomarker levels is unclear (Figure 2). It is possible that workers with the minor allele excreted the absorbed isocyanates more quickly and, thus, could be less susceptible to developing adverse health effects such as isocyanate-induced asthma. Another possibility is that their higher biomarker levels could be in part from delayed excretion from exposures received during the previous day or earlier in the work week. Under this scenario, the workers with homozygous minor allele genotypes could be at a higher risk of adverse health effects. It is possible that our finding that all workers who were homozygous for the minor allele had higher biomarker levels overall is an artifact of the small population size of this study. Nevertheless, this finding should be further investigated and confirmed in future studies. More research is needed to evaluate the implications of homozygosity for minor and major alleles for the workers’ risk of developing isocyanate-induced asthma.

When considering how to implement the results of this study, ethical concerns about using genetic information in worker cohorts have to be kept in mind. However, if confirmed in future studies, the knowledge that genetics impacts isocyanate biomarker levels could be used to protect workers without having to genotype them. For example, more closely monitoring workers with vastly different biomarker levels could be initiated to determine if the increased biomarker levels are due to variation in exposure intensities and/or work practices or if it is due to inherent individual differences (i.e., physiology and genetics). If individual differences seem to be the primary reason for the variation in biomarker levels, then those workers could be monitored more closely to make sure they are adequately protected.

### Linear Mixed-Effects Models

For isocyanate exposure monitoring, linear mixed-effects models results indicated that the skin exposure route is important to monitor in addition to the inhalation route because both routes of exposure influence isocyanate biomarker levels in plasma and urine. The reason for inhalation exposure to HDI monomer being insignificant for HDA urine levels, whereas inhalation exposure to HDI isocyanurate is significant for TAHI urine levels, is unclear. This observation needs further confirmation because it could indicate an important difference in the ADME of HDI monomer versus HDI isocyanurate. The impact of skin exposure on biomarker levels, as we have demonstrated here, can have significant implications. Even though the mechanism of respiratory sensitization is still unknown, there is evidence that exposure to isocyanates can cause respiratory sensitization through skin exposure alone (Beck and Leung, 2000; Aalto-Korte et al., 2012). The sensitizing ability of isocyanate skin exposure combined with our finding that skin exposure has a significant impact on biomarker levels means that it is important for industrial hygienists to monitor and reduce skin exposure in addition to respiratory exposure in order to better protect workers’ health.

### Bioinformatics

Bioinformatics was consistent for the HDI monomer and HDI isocyanurate biomarker levels. The overlap in functions of the biomarker-associated genes for both compounds provides evidence that the toxicokinetics for HDI monomer and HDI isocyanurate is similar to one another. Overlapping functions in GeneMANIA, NCBI, and DAVID for HDA and TAHI biomarker-related genes included cell morphogenesis and migration, transcription regulation, vascular morphogenesis, transmembrane receptor protein serine/threonine kinase activity, regulation of bone mineralization, pathway-restricted SMAD protein phosphorylation, TGF-β signaling pathway, and inflammatory response.

Multiple genes in our analysis are involved in inflammatory pathways. One such pathway that appeared repeatedly in our analysis is TGF-β. Four of the genes associated with isocyanate biomarker levels, three with HDA (*ACVR1*, *ACVR1C*, and *GDNF*), and one with TAHI (*BMPR1B*), are all involved in the TGF-β cytokine pathway. ACVR1, ACVR1C, and GDNF all play a role in the activation of SMAD transcription factors that then increase levels of the cytokine TGF-β (Bondestam et al., 2001; Tao et al., 2019; Valer et al., 2019). The *BMPR1B* gene encodes a protein that is part of the transmembrane serine/threonine kinase family, and its ligands [bone morphogenetic proteins (BMPs)] are members of the TGF-β cytokine superfamily (RefSeq, 2012). Furthermore, GeneMANIA showed that all of the query genes for HDA except *PCARE/C2orf71* and all query genes for TAHI except *CNTN3* have physical or genetic interactions with genes that are involved in the TGF-β pathway through activin, SMAD, and/or BMP signaling (Figures 3, 4). Notably, TGF-β has been demonstrated to be able to affect inflammatory responses in the lung and has been shown to influence airway dysfunction and remodeling in allergic airway disease (Branchett and Lloyd, 2019). Our findings of the importance of the TGF-β pathway after isocyanate exposure are in accordance with research on asthma, in which a genetic marker in TGF-β, rs1800469, has been shown to modulate susceptibility to isocyanate-induced asthma (Yucesoy et al., 2016).

Another inflammation-related gene was *DGKB*, which was associated with HDA levels in urine. *DGKB* regulates diacylglycerol (DAG) concentrations that increase protein kinase C activity (Kamiya et al., 2016), and protein kinase C has been implicated as a contributor to inflammation and bronchoconstriction (Webb et al., 2000; Leppänen et al., 2014; Haick et al., 2017). Although there is a lack of human data on the impact of inflammation on ADME, multiple *in vivo* studies have shown that animals with inflammation have increased tissue uptake of drugs (Vugmeyster et al., 2012). This is at least in part because inflammation makes the endothelial barrier around tissues weaker and increases vascular permeability (Vugmeyster et al., 2012; Mittal et al., 2014). It follows that increased uptake of drugs into the tissues could delay their elimination from the body via blood filtration by the kidney. Therefore, these inflammation-related protein networks may be able to influence plasma and urine biomarker levels indirectly by impacting their ADME.

Another finding, which was made more evident by GeneMANIA, was that calcium regulation networks were associated with both HDA and TAHI via the regulation of bone mineralization by the BMP receptors ACVR1 and BMPR1B, respectively. These results are supportive of findings by Chiung and colleagues, in which they demonstrated that *in vitro* exposure to a related isocyanate, TDI, resulted in elevated calcium levels in the cells (Chiung et al., 2010). They also showed that the increase in calcium triggered the release of the pro-inflammatory cytokine interleukin-4 (IL4) (Chiung et al., 2010). Additionally, research on isocyanate-induced asthma has correlated a calcium-dependent cell adhesion gene, *CDH17*, with isocyanate asthma risk (Yucesoy et al., 2015a; Bernstein et al., 2018). Likewise, calcium-mediated changes in cell adhesions may also be able to impact isocyanate toxicokinetics because calcium has been shown to be critical for the formation and maintenance of cell adhesions (Gao et al., 2000), which strengthen endothelial barrier function by decreasing vascular permeability (Gao et al., 2000). Thus, differences in calcium levels can result in inflammation and/or endothelial barrier dysfunction, which could potentially impact isocyanate biomarker levels by increasing their distribution into tissues and delaying their excretion. The role of calcium should be studied further because it may be important for impacting both the toxicokinetics and toxicodynamics of isocyanates.

Other overlapping functions for HDA and TAHI biomarker-related genes included serine/threonine protein kinase signaling, cell morphogenesis and migration, gene regulation, and vascular morphogenesis. One gene of note in this category from the TAHI urine analysis was the *GRK5* gene, which is part of the serine/threonine protein kinase family, and contributes to regulating the migration of polymorphonuclear leukocytes (PMNs) in response to chemokines (Fan and Malik, 2003). Two other genes, *ETV1* from the HDA urine analysis and *SALL1* from TAHI urine analysis, are genes that encode transcription factors, which regulate multiple processes that also include cell migration as well as angiogenesis (Yamamoto et al., 2010; RefSeq, 2015). The protein product of a third gene, *PDZRN3* from the TAHI urine analysis, may be involved in vascular morphogenesis as well (RefSeq, 2019). The potential impact of these pathways on plasma and urine biomarker levels should be researched in future studies.

The types of genes that contained or were proximal to the significant SNPs included pseudogenes and ncRNA in addition to protein coding genes. Pseudogenes are not translated into protein but can still be transcribed into RNA and can then impact the expression of protein-coding genes such as by forming microRNA (miRNA) or small interfering RNA (siRNA) (Hui et al., 2013; Li et al., 2013; Pink and Carter, 2013; Roberts and Morris, 2013; Groen et al., 2014; Grandér and Johnsson, 2016). ncRNAs are similar to pseudogenes in that they are generally unable to be translated into protein and are regulators of protein expression (Giambruno et al., 2018), although recent evidence suggests that a subset of ncRNA encodes small proteins with < 100 amino acids (Zhu et al., 2018). ncRNAs impact drug metabolism and disposition (Ning et al., 2019), and many studies have linked ncRNA to a wide range of diseases including cardiovascular disease, breast cancer, and liver disease (Hung et al., 2018; Klinge, 2018; Dong et al., 2019; Heo et al., 2019). As such, the significant SNPs within pseudogenes and ncRNA may be impacting the proteome in a way that is important for the toxicokinetics and/or toxicodynamics of HDI monomer and HDI isocyanurate.

Furthermore, the 20 statistically significant SNPs may all have a role in gene regulation regardless of whether they were intronic or intergenic. Recently, it was observed that non-coding SNPs associated with isocyanate-induced asthma can result in allele-dependent changes in gene expression and differential nuclear protein and histone binding (Bernstein et al., 2018). Other studies have shown that intronic SNPs can impact enhancer activity and splicing, which can result in altered mRNA and protein expression and can change the characteristics of those genes (Wang et al., 2014; Wang and Sadee, 2016; Schwartz et al., 2017). Intergenic SNPs have also been observed to impact enhancer activity and the binding of transcription factors (Flora et al., 2013; Spasovski et al., 2013; Antontseva et al., 2015; Schierding et al., 2016). Thus, the intronic and intergenic SNPs we have identified could be impacting biomarker levels after isocyanate exposure by acting as modifiers of gene regulation and transcription.

### Candidate-Gene Analysis

The candidate-gene analysis identified four biomarker-associated SNPs that are within three genes that were reported in a GWAS by Kim and colleagues to have asthma-associated SNPs (Kim et al., 2009) when a MAF > 0.05 was used. The genes and SNPs were *ALK1* (rs6731230 in our study, and rs7599783, rs7599686, and rs4666199 in Kim’s study), which encodes a receptor tyrosine kinase that is activated by TGF-β/BMP and increases the proliferation and migration of endothelial cells (Goumans et al., 2003; Akla et al., 2018); *DOCK2* (rs12522080 and rs12515095 in our study, and rs10054135 in Kim’s study), which is integral for lymphocyte migration in response to chemokines (Sanui et al., 2003; Nishikimi et al., 2013; Chen et al., 2018); and *LHPP* (rs11598732 in our study and rs4962619 in Kim’s study), which encodes a phosphatase that has been reported to induce apoptosis (Zheng et al., 2018; Zhang et al., 2019). These pathways compliment the bioinformatics on the significant biomarker-associated SNPs well, in which there was overlap with the TGF-β pathway (ALK and ACVR1, ACVR1C, GDNF, and BMPR1B), chemokine-induced immune cell motility (DOCK2 and GRK5), and apoptosis regulation (LHPP and GDNF; Zheng et al., 2018; Zhang and Wang, 2019).

Additionally, one of the genes that contains a significant SNP identified in this study, *KCNQ5*, is in the same family as one of the candidate genes, *KCNIP4*, thus lending further evidence to the potential importance of this gene family, which helps regulate airway smooth muscle cell contraction and is important in asthma (Valverde et al., 2011; Kocmalova et al., 2015). In context, the potassium channels (KCN) family has 79 genes. Assuming approximately 20,000 genes in the human genome (Salzberg, 2018), the *KCN* gene family represents about 0.3% of all protein-coding genes. These results can help guide research on identifying pathways for the development of adverse health effects.

### Limitations

The small sample size, which is common for quantitative chemical exposure assessment studies, is the main limitation of this study. Strict QC [i.e., Hardy–Weinberg equilibrium (HWE) < 0.001 and MAF > 0.10] was incorporated owing to the small sample size in order to help make the results more robust. However, there were 10 significant SNPs for which none of the workers were homozygous for the minor allele. Additionally, some of the 20 significant SNPs could be false positives, thus, a larger study population is required to replicate these results. However, despite the small sample size, this study showcases that GWAS can be conducted in small cohorts when quantitative personal exposure and biomarker measurements are available and included in the models. We demonstrate that this methodology increases the power and is particularly useful when it is not possible to recruit large numbers of participants as is often the case in occupational exposure assessment and epidemiology studies.

Another limitation in this study is the potential overestimation or, more likely, underestimation of the inhalation and skin exposure levels to HDI monomer and HDI isocyanurate. All spray painters used some form of respiratory protection, so we adjusted the measured breathing zone concentrations on the basis of the type of respirator worn, which was primarily a half-face respirator worn by the workers (note: workers’ personal protective equipment worn during visits is reported in Supplementary Table S4; Flack et al., 2010b). An estimate of an adult male worker’s breathing rate was also used so that an inhaled dose could be calculated. Because of these estimations, a worker’s true inhalation exposure may differ from these adjusted values. For skin exposure, tape-strip sampling provides a point estimate of the skin exposure through only the first 5 μm of the stratum corneum (Nylander-French, 2000). Thus, any amount that had already penetrated through the first 5–10 cell layers of the stratum corneum was not accounted for in the measured levels of skin exposure. Furthermore, HDI isocyanurate penetrates through the skin more rapidly than does HDI monomer (Thomasen and Nylander-French, 2012), and, therefore, HDI isocyanurate skin exposure measurement is more subject to under-quantification than HDI monomer.

Lastly, urine samples were collected only during the work shift, per the monitoring scheme for HDI monomer biomarker assessment based on its short half-life of about 3 h (Tinnerberg et al., 1995; Liu et al., 2004; Gaines et al., 2010a; Budnik et al., 2011). However, the half-life of HDI isocyanurate is unknown and could be longer than that of the HDI monomer. Thus, the urine TAHI biomarker levels measured in this study may underestimate same-day exposure to HDI isocyanurate. In future studies, determination of the half-life for HDI isocyanurate and making appropriate adjustments in the timing of urine and blood sample collections are needed to improve exposure assessment for HDI isocyanurate.

### Strengths

A strength of this study is the depth of the personal quantitative exposure assessment (i.e., inhalation, skin, and biomarkers of exposure measurements). We were able to use reliable and specific biomarkers to estimate workers’ internal exposure dose by measuring their plasma and urine biomarker levels during the workday. We have previously demonstrated that HDA is excreted in urine with a half-life of ∼2.9 h and that urine HDA levels are correlated with same-day measurements of HDI monomer exposure (Gaines et al., 2010a,b, 2011). For HDI isocyanurate, we have demonstrated that TAHI in urine is a specific biomarker of HDI isocyanurate exposure and that urine TAHI levels are correlated with same-day exposure measurements of HDI isocyanurate exposure (Robbins et al., 2018). Urine biomarker levels were creatinine adjusted in order to account for differences in workers’ hydration levels (Gaines et al., 2010a), and skin exposure values along with plasma levels were adjusted to account for differences in workers’ body sizes (Fent et al., 2009b; Flack et al., 2010b).

Complete industrial hygiene data on exposure and biomarker levels were collected for every worker on up to three different visits at least 3 weeks apart (workers’ personal protective equipment worn during the visits is reported in Supplementary Table S4). Having these multiple measurements per worker makes the industrial hygiene data more robust and less affected by error and bias and, thus, makes the exposure and biomarker associations more reliable. The personal quantitative exposure and biomarker measurements also increased the power in the study (Bush and Moore, 2012) and enabled us to obtain statistically significant results despite the small study sample size. These measures aid in controlling for potentially confounding factors and lend more credibility to the obtained results.

Strengths of the genetics analysis include the prospective nature of GWAS (which is not limited by previous research and hypotheses), the inspection of LocusZoom plots, and the use of GeneMANIA. LocusZoom plots show that nearby sentinel SNPs in LD with the 20 significant SNPs tended to have higher statistical significance than nearby SNPs that were not in LD (Supplementary Figures S1–S3), providing more confidence in the results. Additionally, bioinformatics was based on evaluating genes that contained or were proximal to the 20 significant SNPs, meaning that if the biomarker-associated SNPs we detected are not the true effector SNPs but are in LD with the effector SNPs, then the bioinformatics should be unaffected as long as the biomarker-associated SNPs and effector SNPs are proximal to the same gene(s). The use of GeneMANIA to assess the predicted protein networks for those genes helped to highlight biomarker-associated pathways that may otherwise have been overlooked if the genes had been examined outside of the context of the networks they impact. In this way, GeneMANIA helped to make the overlap between HDA and TAHI biomarker-related gene networks clearer. Furthermore, MDS analysis ruled out smoking and ethnicity as covariates for our study population, the latter of which indicates that population stratification should not be significantly impacting our GWAS results.

## Conclusion

We have demonstrated that genetic polymorphic markers are associated with plasma and urine biomarker levels following exposure to HDI monomer and HDI isocyanurate in automotive spray painters. The 20 significant SNPs have not been previously identified in either candidate-gene or GWAS isocyanate asthma research, and the different genotypes for these SNPs were associated with 4- to 16-fold differences in biomarker levels. We have shown that personal quantitative exposure and biomarker measurements are important to include in order to account for individual variation in GWAS-based exposure assessment and epidemiology studies. Additionally, our mixed models showed that both inhalation and skin exposures have a significant impact on isocyanate biomarker levels, indicating that it is important to prevent not only occupational inhalation exposure but also skin exposure to isocyanates.

In the bioinformatics analyses, we found that transcription regulation, calcium transport, vascular morphogenesis, and the TGF-β pathway (which has previously been associated with impacting isocyanate-induced asthma susceptibility) might alter isocyanate toxicokinetics, potentially indirectly by impacting inflammation levels in the body. We also identified biomarker-associated SNPs that were within three candidate genes that contain SNPs which have been associated with isocyanate-induced asthma (*ALK1*, *DOCK2*, and *LHPP*), demonstrating that there may be overlapping protein networks that impact both the toxicokinetics and toxicodynamics of isocyanates. The high turnover rate of workers in the automotive industry made it infeasible for us to incorporate a longitudinal component on the health outcomes of these workers. However, this study helps fill an important knowledge gap about the biological pathways involved in isocyanate metabolism and excretion and before adverse health outcomes develop.

If the genetic markers that we identified are verified with more research, then additional bioinformatics and experimental studies in appropriate *in vitro* and *in vivo* models are needed to investigate the biological plausibility of these associations. Moreover, other quantitative exposure assessment studies that include GWAS component are needed to investigate the relationships between these 20 biomarker-associated SNPs, their proximal genes, and their potential impact on toxicokinetics and health effects associated with isocyanate exposures, including isocyanate-induced asthma.

## Data Availability Statement

The datasets for this article are not publicly available due to concerns regarding participant/patient anonymity. Requests to access the datasets should be directed to the corresponding author.

## Ethics Statement

The study was approved by The University of North Carolina at Chapel Hill Office of Human Research Ethics Institutional Review Board (Study #12-1195) and by the Washington State Department of Social and Health Services Institutional Review Board (Study #A-013106). The patients/participants provided their written informed consent to participate in this study.

## Author Contributions

LT designed and carried out the model computations, created the figures, performed the bioinformatics analyses, and wrote the manuscript. JF designed and provided guidance for the genetics and bioinformatics methods. ZR conducted the quantitative analysis of the exposure and biomarker levels. JB prepared the DNA samples for genotyping. LN-F designed and supervised the project. All authors contributed to the preparation of the final manuscript and approved it for publication.

## Conflict of Interest

The authors declare that the research was conducted in the absence of any commercial or financial relationships that could be construed as a potential conflict of interest.

## Footnotes

1 https://ldlink.nci.nih.gov/?tab=ldpair

2 http://www.internationalgenome.org

## Abbreviations

ADME: Absorption, distribution, metabolism and elimination
APF: Assigned protection factor
DAVID: Database for Annotation, Visualization and Integrated Discovery
FDR: False discovery rate
GWAS: Genome-wide association study
HDA: 1,6-diaminohexane
HDI: 1,6-hexamethylene diisocyanate
LD: linkage disequilibrium
LOD: limit of detection
MAF: minor allele frequency
MDL: method detection limit
PBMCs: peripheral blood mononuclear blood cells
QC: quality control
rsID: reference SNP cluster identification
SNP: single-nucleotide polymorphism
TAHI: trisaminohexyl isocyanurate.
