# Supplementary material for: Influence of Genetic Variance on Biomarker Levels After Occupational Exposure to 1,6-Hexamethylene Diisocyanate Monomer and 1,6-Hexamethylene Diisocyanate Isocyanurate
Source: Front Genet. 2020 Aug 19;11:836. doi: 10.3389/fgene.2020.00836 (PMC7466756; doi:10.3389/fgene.2020.00836)

Supplemental Information

Table S1 provides information on the ten most influential single-nucleotide polymorphisms (SNPs) for trisaminohexyl isocyanurate (TAHI) biomarker levels in plasma. Tables S2 and S3 provide details on the linear mixed models. Table S4 describes the personal protective equipment worn by workers during painting tasks. Figures S1, S2, and S3 show LocusZoom plots for the 20 SNPs associated with isocyanate biomarker levels.

Table S1. Genes proximal to ten most influential single-nucleotide polymorphisms (SNPs) for trisaminohexyl isocyanurate (TAHI) biomarker levels in plasma, none of which reached statistical significance (false discovery rate, FDR >0.10). 1,6-Hexamethylene diisocyanate (HDI) isocyanurate skin and inhalation exposures were used as covariates.

SNP	Chr	Position	MAF	p-value	FDR	Proximal Genes
rs11074737	16	25980931	0.18	1.33E-06	0.365	HS3ST4 (intronic)
rs12360058	10	50373347	0.09	1.37E-06	0.365	SGMS1 (intronic)
rs5995123	22	35588003	0.10	2.90E-06	0.411	LOC107985590 (intronic)
rs11001444	10	52282796	0.31	3.08E-06	0.411	PRKG1 (intronic)
rs10842230	12	23827048	0.24	5.23E-06	0.558	SOX5 (intronic)
rs12151356	19	35690144	0.18	1.45E-05	0.994	UPK1A and ZBTB32 (intergenic)
rs10253145	7	147084888	0.20	1.49E-05	0.994	CNTNAP2 and LOC101928700 (intronic)
rs2437775	8	96534295	0.31	1.80E-05	0.994	SDC2 (intronic)
rs7802599	7	149744229	0.30	2.36E-05	0.994	KRBA1 and ZNF467 (intergenic)
rs14983	11	102520694	0.13	3.85E-05	0.994	MMP7 (intronic)

Chr = chromosome; MAF = major allele frequency

Table S2. Linear mixed models with and without the most influential single-nucleotide polymorphism (SNP) for 1,6-hexamethylene diisocyanate (HDI) monomer exposure-adjusted 1,6-diaminohexane (HDA) levels in plasma and urine (p-value <0.10 was considered statistically significant).

A) HDA in plasma.

Effect	Estimate	Standard Error	p-value
Intercept	-2.1497	0.5576	0.0005
HDI Monomer Inhalation Exposure Dose	0.0994	0.1439	0.4930
HDI Monomer Skin Exposure Dose	0.1158	0.0638	0.0758

B) HDA in plasma with most significant SNP.

Effect	Estimate	Standard Error	p-value
Intercept	0.1971	0.6740	0.7719
HDI Monomer Inhalation Exposure Dose	0.2221	0.1266	0.0859
HDI Monomer Skin Exposure Dose	0.0203	0.0604	0.7390
rs2061660, GG (major allele)	-3.2418	0.7482	0.0002
rs2061660, TG; reference	0	.	.

C) HDA in urine.

Effect	Estimate	Standard Error	p-value
Intercept	-2.7163	0.4685	<0.0001
HDI Monomer Inhalation Exposure Dose	0.0763	0.1216	0.5333
HDI Monomer Skin Exposure Dose	0.1592	0.0503	0.0026

D) HDA in urine with most significant SNP.

Effect	Estimate	Standard Error	p-value
Intercept	0.1919	1.0263	0.8530
HDI Monomer Inhalation Exposure Dose	-0.0593	0.1206	0.6251
HDI Monomer Skin Exposure Dose	0.1833	0.0467	0.0003
rs489332, CC (major allele)	-3.6576	1.0692	0.0019
rs489332, CT	-2.1742	1.2096	0.0831
rs489332, TT; reference	0	.	.

Table S3. Linear mixed models with and without the most influential single-nucleotide polymorphism (SNP) for 1,6-hexamethylene diisocyanate (HDI) isocyanurate exposure-adjusted trisaminohexyl isocyanurate (TAHI) levels in plasma and urine (p-value <0.10 was considered statistically significant).

A) TAHI in plasma.

**Effect**	**Estimate**	**Standard Error**	**p-value**
Intercept	-7.2683	0.6154	<0.0001
HDI Isocyanurate Inhalation Exposure Dose	0.1433	0.1424	0.3192
HDI Isocyanurate Skin Exposure Dose	0.0513	0.0883	0.5638

B) TAHI in urine.

**Effect**	**Estimate**	**Standard Error**	**p-value**
Intercept	-8.6538	0.6507	<0.0001
HDI Isocyanurate Inhalation Exposure Dose	0.3016	0.1483	0.0469
HDI Isocyanurate Skin Exposure Dose	0.1693	0.09405	0.0775

C) TAHI in urine with most significant SNP.

**Effect**	**Estimate**	**Standard Error**	**p-value**
Intercept	-8.9307	0.6236	<0.0001
HDI Isocyanurate Inhalation Exposure Dose	0.2955	0.1400	0.0395
HDI Isocyanurate Skin Exposure Dose	0.1623	0.0919	0.0808
rs1866929, CC	1.5681	2.0129	0.4421
rs1866929, TC	3.2917	1.1644	0.0083
rs1866929, TT; reference (major allele)	0	.	.

Table S4. Personal protective equipment worn by the 33 workers during all 88 monitored visits.

Respirator Type	Half-face respirator	Full-face respirator with cartridges	Full-face or hood respirator with air-supply hose	Hood with a powered air-purifying respirator
% of Visits	74%	3%	15%	8%

Other PPE	Coveralls	Gloves	Hat	Goggles
% of Visits PPE was worn	72%	82%	39%	18%

Figure S1. LocusZoom plots of the seven significant single-nucleotide polymorphisms (SNPs) for 1,6-diaminohexane (HDA) levels in plasma, showing nearby SNPs in linkage disequilibrium (LD) and showing proximal genes.

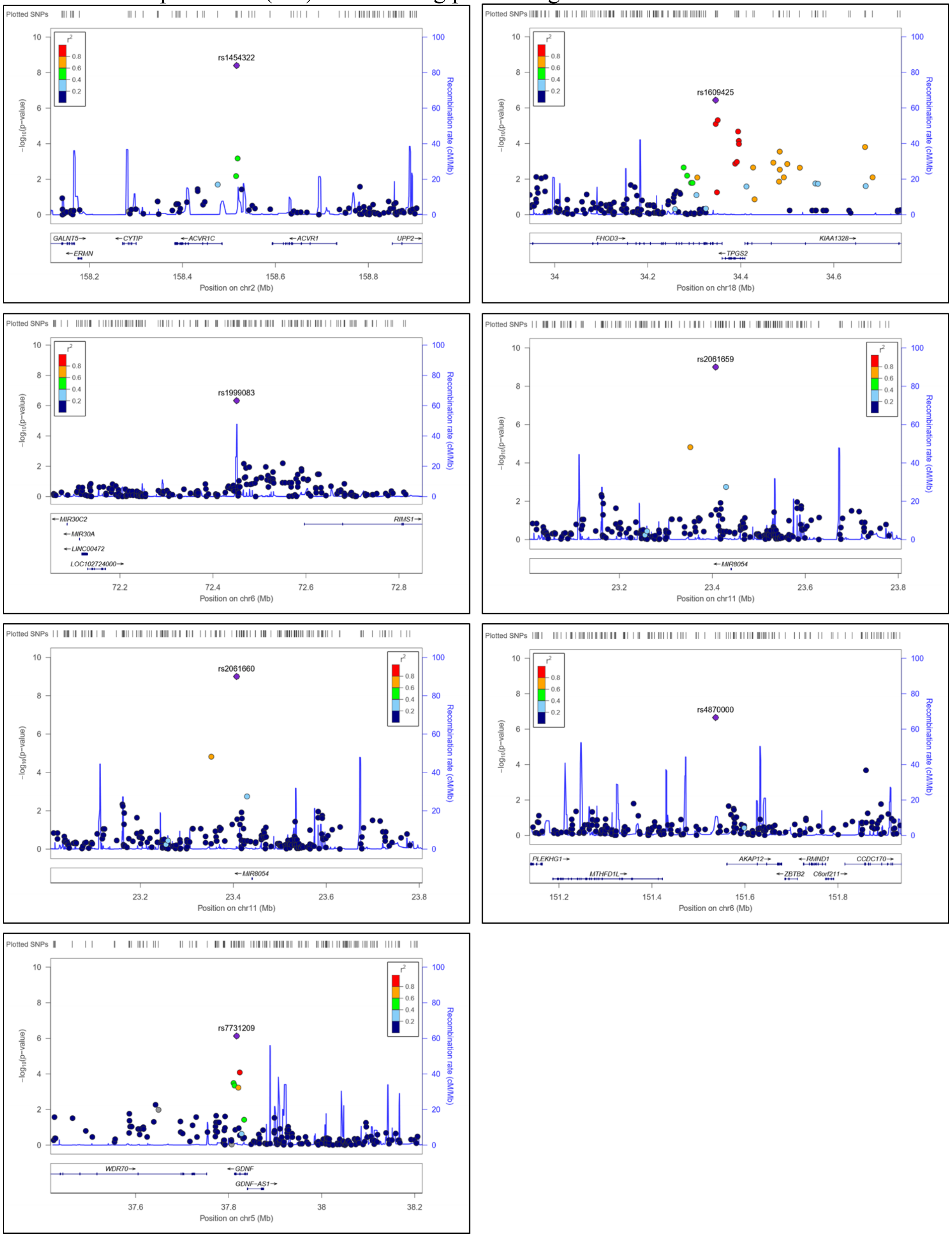

Figure S2. LocusZoom plots of the five significant single-nucleotide polymorphisms (SNPs) for 1,6-diaminohexane (HDA) levels in urine, showing nearby SNPs in linkage disequilibrium (LD) and showing proximal genes.

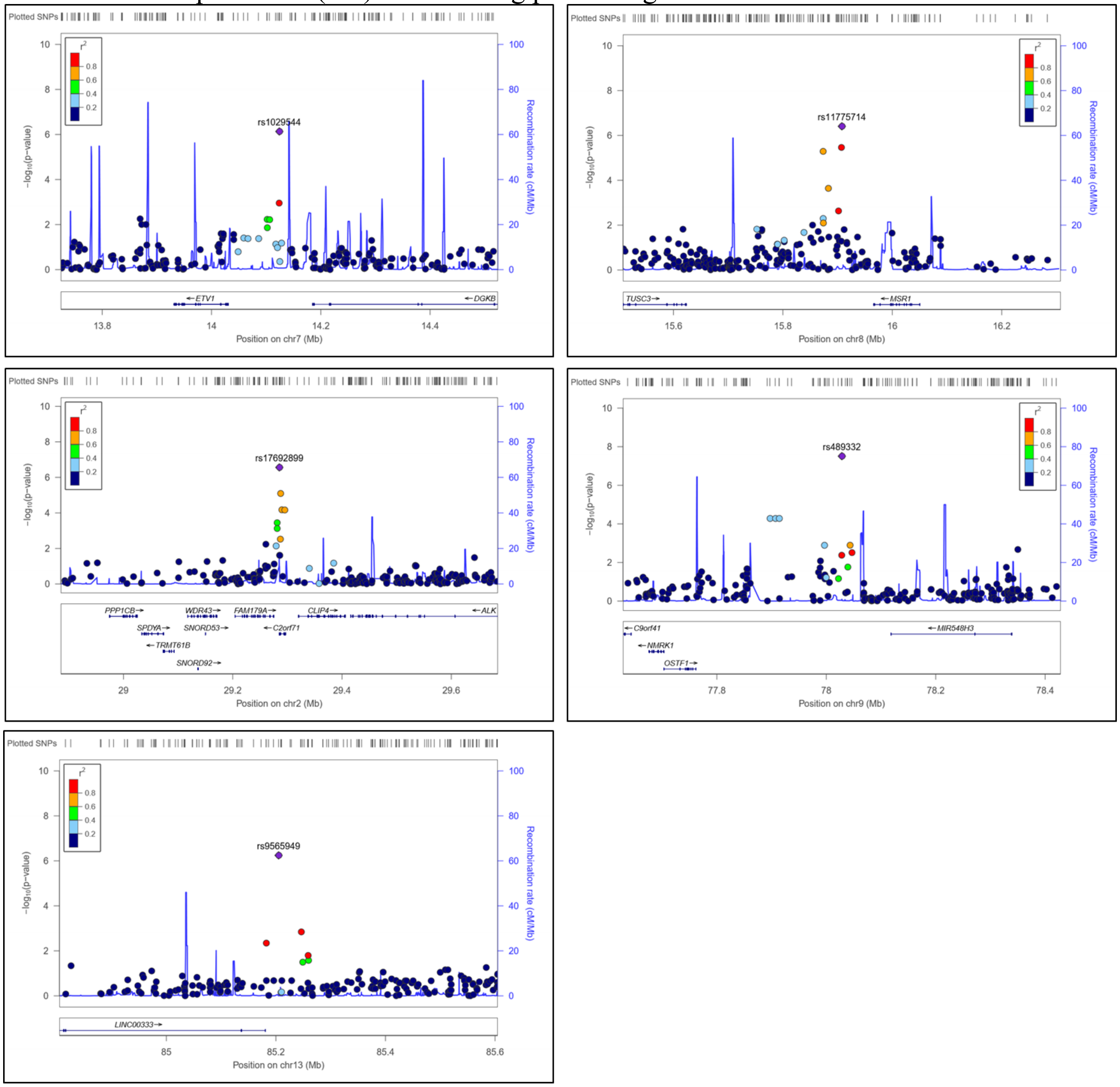

Figure S3. LocusZoom plots of the eight significant single-nucleotide polymorphisms (SNPs) for trisaminohexyl isocyanurate (TAHI) levels in plasma, showing nearby SNPs in linkage disequilibrium (LD) and showing proximal genes.

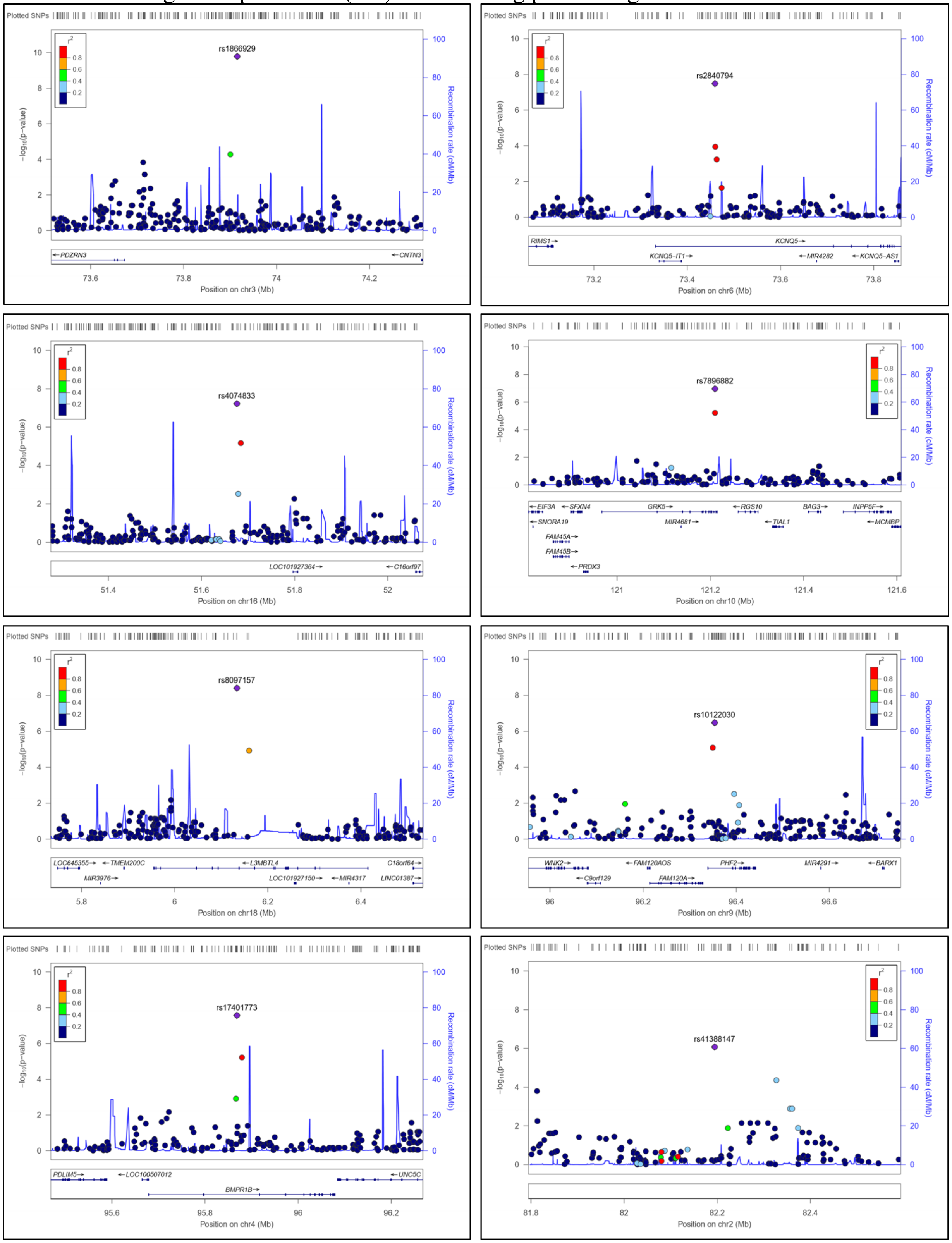

Supplement: Supplementary file 1 [file Data_Sheet_1.pdf]
